# HEAT EXPOSURE AND ADAPTIVE IMMUNITY AMONG OLDER US ADULTS

**DOI:** 10.1093/geroni/igae098.1007

**Published:** 2024-12-31

**Authors:** Eunyoung Choi, Gokul Seshadri, Jennifer Ailshire, Bharat Thyagarajan

**Affiliations:** University of Southern California, Los Angeles, California, United States; University of Minnesota, Minneapolis, Minnesota, United States; University of Southern California, Los Angeles, California, United States; University of Minnesota, Minneapolis, Minnesota, United States

## Abstract

Climate change is leading to more extreme heat events, potentially impacting host-parasite dynamics, immunity, and infectious diseases. While animal studies suggest that heat can induce long-lasting immune disorders, its effects on human immune function remain underexplored. This study examines how heat exposure is associated with adaptive immunity, focusing on T-and B-cell subsets in a national sample of older adults from the 2016 Health and Retirement Study Venous Blood Study (N=7,301). Using high-resolution meteorological data from gridMet, daily heat index values were calculated at the census tract level and linked to each respondent’s residential location. We examined both acute (day of blood collection) and chronic (1-year average) heat exposure using multilevel models, adjusting for individual factors (e.g., age, CMV seropositivity). For acute exposure, higher heat index values (standardized) were associated with decreased proportions of CD4+ naïve T-cells (B:-0.01;p<.001), CD8+ memory T-cells (B:-0.002;p<.05), and IgD- memory B cells (B:-0.003;p<.01), but increases in Naïve B cells (B:0.01;p<.001). For chronic exposure, higher heat index values were associated with decreased proportions of CD4+ naïve T-cells (B:-0.01,p<.01) and CD8+ memory T-cells (B:-0.002,p<.05). These findings show that heat stressors can affect the immune cells and subsequent cellular and humoral immune responses. Chronic heat stress potentially leads to a reduction in T-cell repertoire that may result in poor adaptive immunity. Acute heat stress may prompt both suppressive and proliferative responses in different cell types. This study underscores the need for further research to assess the broader implications of climate change on the immune health of older populations.

